# Impact of Pre-Hospital Intravenous Infusion on Physiological Parameters in Severe Trauma Patients

**DOI:** 10.7759/cureus.71770

**Published:** 2024-10-18

**Authors:** Hirofumi Mishima, Koshi Nakagawa, Hidekazu Takeuchi, Hiroyuki Takahashi, Shusuke Saito, Shuji Sakanashi, Daizoh Saitoh, Hiroshi Takyu, Hideharu Tanaka

**Affiliations:** 1 Graduate School of Emergency Medical System, Kokushikan University, Tokyo, JPN; 2 Tokyo Fire Department, Nerima Fire Station, Tokyo, JPN; 3 Department of Integrated Science and Engineering for Sustainable Societies, Faculty of Science and Engineering, Chuo University, Tokyo, JPN; 4 Department of Sports and Medical Science, Faculty of Physical Education, Kokushikan University, Tokyo, JPN

**Keywords:** ambulances, emergencies, injuries, intravenous infusion, shock

## Abstract

Introduction

The relationship between pre-hospital intravenous (IV) infusions administered by emergency life-saving technicians (ELSTs) to trauma patients in shock and the resulting variability in their vital signs before hospital arrival remains unclear. In 2014, Japan approved the use of lactated Ringer’s solution via IV by ELSTs for patients aged 15 and older with non-cardiac arrest and shock symptoms not caused by cardiogenic factors. However, the impact of pre-hospital IV infusions on physiological parameters in severely injured trauma patients is still unknown.

Aim

The aim of this study is to investigate the impact of pre-hospital IV infusions administered by ELSTs on trauma patients with shock, focusing on the resulting variations in the shock index and other physiological parameters in the pre-hospital setting.

Methods

This retrospective cohort study included patients registered in the Japan Trauma Data Bank who were transported by ambulance from the pre-hospital to the hospital by ELST between 2019 and 2021. First, the data were categorized based on pre-hospital IV access as either IV (+) or IV (-). Propensity score matching was then performed to estimate the average treatment effect for patients receiving IV (+). The primary endpoint was the delta shock index (DSI), while secondary endpoints included systolic blood pressure (sBP), heart rate (HR), and respiratory rate (RR). Welch’s t-test was used to estimate mean differences and 95% CIs, and Cohen’s d was calculated to measure effect sizes.

Results

A total of 88,817 patients were enrolled in the study, with 19,793 included in the analysis. Of these, 778 patients were matched for comparison. IV access (+) was not significantly associated with changes in the DSI, showing a small effect size (-0.09 vs. -0.06; difference [95% CI]: -0.04 [-0.08 to 0.00]). Additionally, IV (+) was not significantly associated with differences in HR (-0.23 vs. 1.16; difference [95% CI]: -1.40 [-3.59 to 0.80]) or RR (-1.95 vs. -1.08; difference [95% CI]: -0.87 [-1.83 to 0.09]), both of which demonstrated small effect sizes. However, IV (+) was significantly associated with an increase in sBP difference, although the effect size remained small (13.22 vs. 8.73; difference [95% CI]: 4.49 [0.35 to 8.62]).

Conclusions

IV access was not directly associated with variations in the shock index in the pre-hospital setting; however, it significantly increased sBP. Future studies should include the volume of IV infusion to further elucidate these findings.

## Introduction

Traumatic death is a significant public health concern globally [1,2]. In Japan, it ranks as a leading cause of mortality among individuals under 40 years old and substantially contributes to deaths in young and middle-aged populations [3]. In 2003, the Japan Advanced Trauma Evaluation and Care Council for Physicians and the Japan Pre-hospital Trauma Evaluation and Care (JPTEC) Council for Emergency Medical Services (EMS) were established to reduce, and ultimately eliminate, preventable trauma deaths.

The relationship between pre-hospital intravenous (IV) infusions administered by emergency life-saving technicians (ELSTs) to trauma patients in shock and the variability in these patients' vital signs prior to hospital arrival remains unclear. Previous research has indicated inconsistent effects of IV administration on survival and mortality rates among trauma patients [4-9]. In 2014, Japan approved the administration of lactated Ringer’s solution via IV by ELSTs for patients aged 15 and older who exhibit shock symptoms not due to cardiogenic causes [10]. However, the physiological variations resulting from pre-hospital IV administration by ELSTs in severely injured patients are still unknown. The shock index is recognized as a valuable indicator of shock severity and outcomes [11-13]. Therefore, this study aims to investigate the impact of pre-hospital IV administration by ELSTs on physiological parameters in trauma patients with shock, with a particular focus on the shock index.

## Materials and methods

Study design

This retrospective cohort study utilized data obtained from the Japan Trauma Data Bank (JTDB). The study received approval from the Ethics Committee of Kokushikan University (approval number 23020).

Study setting

Japan has a national land area of approximately 378,000 km² and a total population of about 125 million, with 29% aged 65 years or older [14]. EMS operate 24 hours a day, provided by the fire department. Each ambulance is typically staffed by three EMS personnel, including at least one ELST. Established in 1991, ELSTs are authorized to perform advanced airway management and administer IV adrenaline in cases of cardiac arrest under the online supervision of a medical director. As of April 2014, ELSTs have also been authorized to administer IV fluids to patients experiencing non-cardiac arrest shock [10].

The decision to perform IV therapy, including the infusion rate and volume, is guided by the online direction of a medical doctor, as stipulated by regional medical control (MC) councils. The law mandates that only ELSTs who have completed specialized training in advanced life support - covering the pathophysiology of shock and practical skills such as IV administration - and have been certified by their regional MC council may perform IV therapy on patients aged 15 years or older who are at risk of life-threatening trauma or endogenous disease.

Certified ELSTs can infuse lactated Ringer’s solution through a peripheral venous line with remote medical instructions. The decision to initiate IV therapy is based on an assessment of the patient’s physiological parameters, including blood pressure, level of consciousness, and skin condition. Following a medical doctor’s instructions, the ELST secures a peripheral venous line and begins the infusion per the prescribed volume and rate. The infusion rate is adjusted according to the patient’s blood pressure and the amount of bleeding, with any changes in volume at the doctor’s discretion. However, as a protocol, ELSTs do not determine the infusion volume.

EMS protocols are established independently by regional MC councils under directives from the Ministry of Health, Labor, and Welfare, as well as the Fire and Disaster Management Agency in Japan. Regarding IV protocols, each ELST evaluates whether the patient is in shock and receives remote instructions from the doctor about administering IV therapy and the corresponding infusion volume. There are slight variations depending on the policies of each MC council and the medical facility affiliated with the doctor. For example, measures to control excessive fluid infusion are left to the discretion of the remote physician, leading to a lack of unified IV protocols at the national level, which may result in an incomplete pre-hospital IV protocol in Japan.

The ELST assesses the severity and urgency of a patient’s condition, transporting those with mild to moderate conditions to the nearest level 2 emergency hospital and those with more severe conditions to the nearest level 1 emergency and critical care center. Pre-hospital emergency medicine for trauma in Japan, guided by the JPTEC, emphasizes the rapid transport of patients to the appropriate facility [3]. The mission of the ELST is to prevent the deterioration of patients’ conditions.

Data collection and quality control

The Japanese Association for Acute Medicine and the Japanese Association for the Surgery of Trauma established the JTDB as an academic case registration system aimed at enhancing the quality of trauma care in Japan. Data collection began in 2004, with each participating medical institution registering cases that have an Abbreviated Injury Scale (AIS) score of ≥3. As of March 2022, a total of 303 hospitals and research institutions were involved in the initiative [15]. The registered data encompass initial patient information, transfer details, pre-hospital data, emergency department examinations and treatments, injury severity, as well as admission and discharge information, totaling 92 data items. All analyses were conducted using the information registered in the JTDB.

Selection of participants

The study included patients admitted to hospitals between January 1, 2019, and December 31, 2021, with specific inclusion criteria requiring that trauma patients were transported directly from the pre-hospital setting to the hospital by ambulance with onboard ELSTs. Exclusion criteria encompassed any pre-hospital contact with a physician (as physicians can administer hemostatic agents and blood transfusions), cardiac arrest in the pre-hospital setting or upon hospital arrival, unrealistic values recorded in either setting due to potential errors, unknown pre-hospital emergency treatments, patients under 15 years of age, injuries not classified as penetrating or blunt trauma (such as burns or unclassified injuries), severe single-head trauma indicated by Cushing’s sign (which can influence blood pressure and pulse rate), transport times outside the range of 1 to 60 minutes, and extreme vital sign values (systolic blood pressure (sBP) <60 or ≥241 mmHg; heart rate (HR) <40 or ≥151 beats/min; respiratory rate (RR) <10 or ≥51 counts/min; pre-hospital oxygen saturation <60%). Through list-wise deletion, all missing data (23,659 patients, 26.6%) and cases not meeting eligibility criteria (45,365 patients, 51.1%) were excluded from the analysis.

Endpoint

The primary endpoint of the study was the delta shock index (DSI), while the secondary endpoints focused on differences in sBP, HR, and RR. The DSI was calculated as the difference between the shock index at hospital arrival and that recorded in the pre-hospital setting (i.e., hospital arrival shock index minus pre-hospital shock index). An increase in DSI exceeding 0.1 has been associated with adverse outcomes, including increased mortality [13,16-19]. Moreover, DSI has proven valuable in identifying patients with occult hypoperfusion [14,18]. Differences in sBP, HR, and RR were calculated by subtracting the pre-hospital values from those recorded at hospital arrival (i.e., hospital arrival value minus pre-hospital value).

Data definitions

The study defines several variables, including transport time categorized into tertiles: 1-9 minutes, 10-15 minutes, and 16-61 minutes. Pre-hospital vital signs are evaluated based on severity and urgency [20,21], with specified ranges for sBP (60-89 mmHg, 90-200 mmHg, 201-240 mmHg), HR (40-49 beats/min, 50-120 beats/min, 121-150 beats/min), RR (10-29 breaths/min, 30-50 breaths/min), and oxygen saturation (60-89%, 90-100%). The level of consciousness is assessed using the Japan Coma Scale (JCS), which includes categories: clear (0), class I (1-3), class II (10-30), and class III (100-300). The shock index is set at a critical threshold of 1.0, which is important for determining shock and mortality [22]. Additionally, the injury severity score (ISS) is established at 15 points, serving as a threshold for assessing injury severity. Body injury areas were classified into nine categories based on AIS scores.

Statistical analysis

Regarding patient characteristics, categorical variables are presented as numbers (percentages) and continuous variables as means with standard deviations. Initially, the data were categorized into two groups based on whether IV therapy was administered in the pre-hospital setting (IV (+) or IV (-)). Subsequently, propensity score matching (PSM) was conducted to estimate the average treatment effect in the treated (ATT) for IV (+) patients. The propensity score (PS) was derived using multivariable logistic regression analysis, with IV (+) as the primary exposure. The model included variables such as age, sex, trauma classification, mechanism of injury, transport time, interventions by ELSTs (including oxygen inhalation, spinal motion restriction, hemostasis and fixation, and airway management), pre-hospital vital signs, ISS, and specific injuries (head, face, neck, thorax, abdomen and pelvis, spine, upper extremity, lower extremity, and body surface). The receiver operating characteristic curve for the logistic model was 0.86, indicating its adequacy for PSM [23]. The nearest neighbor one-to-one PSM without replacement, utilizing a caliper width of 0.2, was employed for the matching process [24]. Levene’s test confirmed the equality of variance, showing heteroscedasticity in the cohort before matching and homoscedasticity after matching. Welch’s t-test was used to estimate mean differences and 95% CIs. Cohen’s d was calculated to measure effect sizes following matching, and the association between IV therapy and endpoints was assessed. In a supplementary analysis, inverse probability weighting (IPW) was evaluated using the same PS to examine the robustness of our findings against changes in patient eligibility and statistical methods. The statistical approaches for this analysis were consistent with those of the primary analysis. The significance level was set at 0.05 (two-tailed) for all analyses. JMP Pro 15.0.0 (SAS Institute, Cary, NC, USA) was utilized for statistical analyses, while R (version 4.2.1; R Foundation for Statistical Computing, Vienna, Austria) was employed to compute the standardized mean differences (SMD) and IPW.

## Results

**Figure 1:**
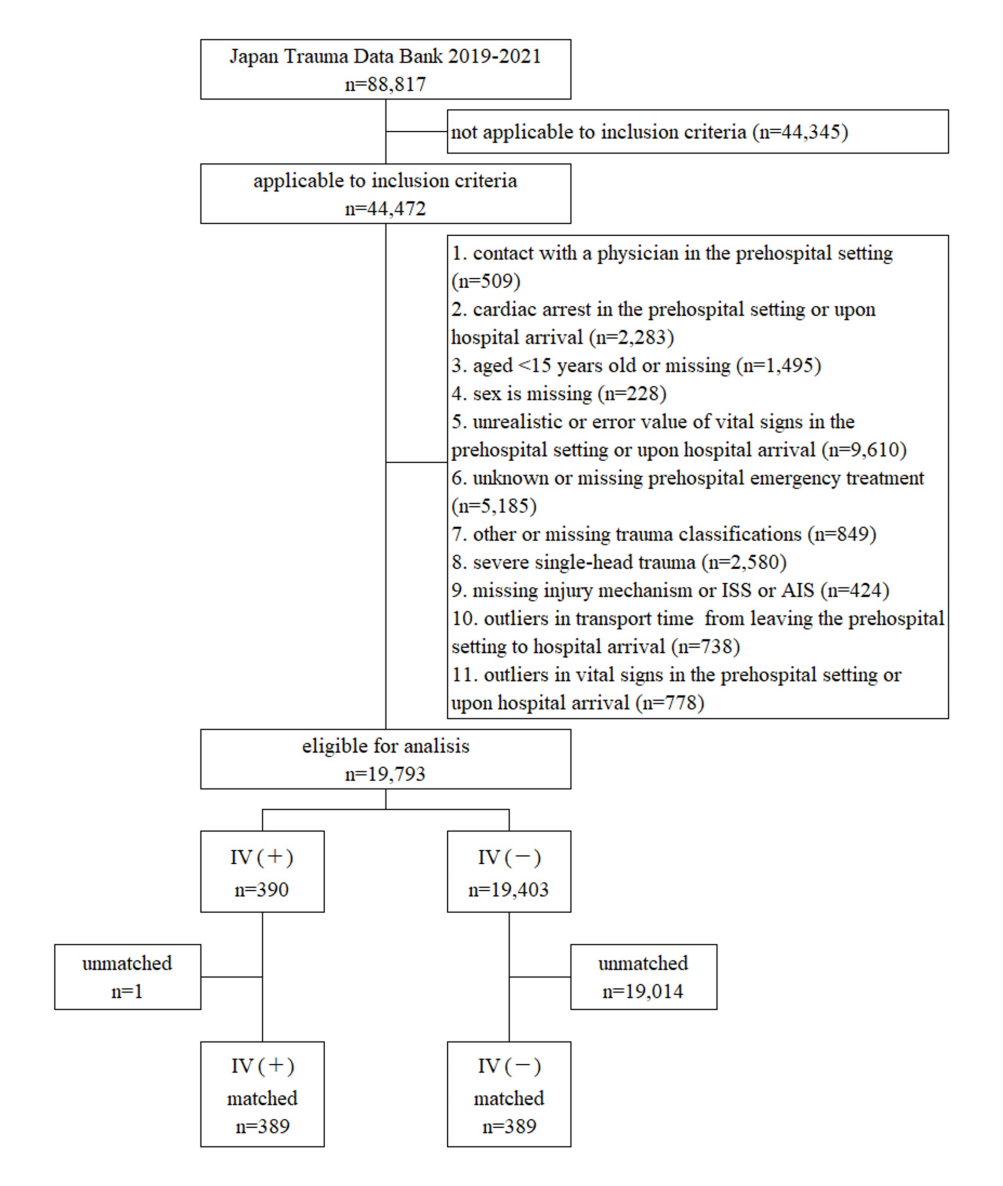
Flowchart for patient enrollment for this analysis AIS, Abbreviated Injury Scale; ISS, injury severity score; IV, intravenous

The data extraction process utilized in this study is illustrated in Figure 1. Throughout the study period, a total of 88,817 patients were registered in the JTDB, of which 19,793 met the eligibility criteria for analysis.

**Figure 2:**
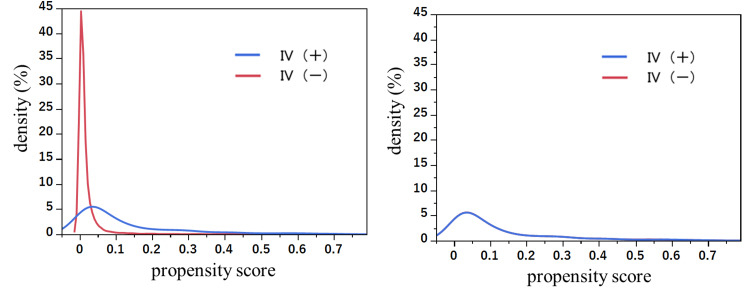
Comparison of PS densities for pre-hospital IV before and after PSM IV, intravenous; PS, propensity score; PSM, propensity score matching

Patient characteristics are summarized in Table 1. Prior to PSM, patients in the IV(+) group displayed distinct characteristics compared to those in the IV(-) group: a higher proportion of patients had a pre-hospital sBP of <90 mmHg (IV(+) vs. IV(-): 36.9% vs. 3.9%), a higher ISS of ≥15 (IV(+) vs. IV(-): 55.6% vs. 28.2%), and longer transport times of ≥16 minutes (approximately 60%) (IV(+) vs. IV(-): 57.4% vs. 31.8%). A total of 778 datasets (389 matched) were extracted based on the PS in both groups. Figure 2 illustrates the density of PSs for IV(+) and IV(-) comparisons before and after PSM. Following PSM, the density of PSs indicated improved approximation and balance between the groups, as demonstrated by the SMD for all variables used in the PS estimate being less than 0.1.

**Table 1:** Patient characteristics with and without IV before and after PSM ELST, emergency life-saving technician; HR, heart rate; ISS, injury severity score; IV, intravenous; JCS, Japan Coma Scale; PSM, propensity score matching; RR, respiratory rate; sBP, systolic blood pressure; SMD, standardized mean difference

Variables	Before matching					After matching				
	IV(+)		IV(-)		SMD	IV(+)		IV(-)		SMD
	n = 390 (%)		n = 19,403 (%)			n = 389 (%)		n = 389 (%)		
Age					0.214					0.052
15-64	219	(56.20)	8,833	(45.50)		219	(56.30)	209	(53.70)	
≥65	171	(43.80)	10,570	(54.50)		170	(43.70)	180	(46.30)	
Sex: male	270	(69.20)	11,648	(60.00)	0.193	270	(69.40)	283	(72.80)	0.074
Trauma classification					0.314					0.047
Blunt injury	345	(88.50)	18,744	(96.60)		344	(88.40)	338	(86.90)	
Penetrating injury	45	(11.50)	659	(3.40)		45	(11.60)	51	(13.10)	
Mechanism of injury					0.468					0.027
Traffic accident	176	(45.10)	7,260	(37.40)		176	(45.20)	179	(46.00)	
Fall	134	(34.40)	10,533	(54.30)		134	(34.40)	129	(33.20)	
Other	80	(20.50)	1,610	(8.30)		79	(20.30)	81	(20.80)	
Transport time (min)					0.544					0.072
1-9	81	(20.80)	7,363	(37.90)		81	(20.80)	74	(19.00)	
10-15	85	(21.80)	5,876	(30.30)		85	(21.90)	96	(24.70)	
16-61	224	(57.40)	6,164	(31.80)		223	(57.30)	219	(56.30)	
Oxygen inhalation by ELST	319	(81.80)	8,167	(42.10)	0.896	318	(81.70)	322	(82.80)	0.027
Spinal motion restriction by ELST	239	(61.30)	9,562	(49.30)	0.243	239	(61.40)	251	(64.50)	0.064
Hemostasis and fixed by ELST	29	(7.40)	2,094	(10.80)	0.117	28	(7.20)	30	(7.70)	0.02
Airway management by ELST	14	(3.60)	197	(1.00)	0.172	14	(3.60)	15	(3.90)	0.014
Pre-hospital sBP (mmHg)					0.921					0.042
60-89	144	(36.90)	760	(3.90)		143	(36.80)	143	(36.80)	
90-199	244	(62.60)	17,880	(92.20)		244	(62.70)	245	(63.00)	
200-240	2	(0.50)	763	(3.90)		2	(0.50)	1	(0.30)	
Pre-hospital HR (beats/min)					0.281					0.074
40-49	3	(0.80)	105	(0.50)		3	(0.80)	6	(1.50)	
50-119	335	(85.90)	18,270	(94.20)		334	(85.90)	329	(84.60)	
120-150	52	(13.30)	1,028	(5.30)		52	(13.40)	54	(13.90)	
Pre-hospital RR (counts/min)					0.505					0.035
10-29	281	(72.10)	17,666	(91.00)		281	(72.20)	287	(73.80)	
30-50	109	(27.90)	1,737	(9.00)		108	(27.80)	102	(26.20)	
Pre-hospital JCS					0.566					0.054
0	111	(28.50)	9,934	(51.20)		111	(28.50)	106	(27.20)	
1-3	162	(41.50)	7,209	(37.20)		161	(41.40)	171	(44.00)	
10-30	58	(14.90)	1,190	(6.10)		58	(14.90)	57	(14.70)	
100-300	59	(15.10)	1,070	(5.50)		59	(15.20)	55	(14.10)	
Pre-hospital oxygen saturation (%)					0.311					0.046
60-89	53	(13.60)	918	(4.70)		52	(13.40)	46	(11.80)	
90-100	337	(86.40)	18,485	(95.30)		337	(86.60)	343	(88.20)	
Pre-hospital shock index					0.744					0.077
<1.0	259	(66.40)	18,267	(94.10)		259	(66.60)	273	(70.20)	
≥1.0	131	(33.60)	1,136	(5.90)		130	(33.40)	116	(29.80)	
ISS: ≥15	217	(55.60)	5,478	(28.20)	0.578	216	(55.50)	220	(56.60)	0.021
Head injury	126	(32.30)	5,980	(30.80)	0.032	126	(32.40)	127	(32.60)	0.005
Face injury	69	(17.70)	3,319	(17.10)	0.015	69	(17.70)	70	(18.00)	0.007
Neck injury	8	(2.10)	279	(1.40)	0.047	8	(2.10)	6	(1.50)	0.039
Thorax injury	164	(42.10)	5,670	(29.20)	0.27	164	(42.20)	150	(38.60)	0.073
Abdomen and pelvis injury	103	(26.40)	1,707	(8.80)	0.475	103	(26.50)	97	(24.90)	0.035
Spine injury	123	(31.50)	5,183	(26.70)	0.106	123	(31.60)	140	(36.00)	0.092
Upper extremity injury	127	(32.60)	5,077	(26.20)	0.141	127	(32.60)	129	(33.20)	0.011
Lower extremity injury	191	(49.00)	9,531	(49.10)	0.003	190	(48.80)	188	(48.30)	0.01
Body surface injury	24	(6.20)	874	(4.50)	0.073	24	(6.20)	27	(6.90)	0.031

Table 2 presents the results of the analysis. The administration of IV therapy (IV(+)) was not significantly associated with changes in the DSI, with a small effect size observed (-0.09 vs. -0.06; difference [95% CI]: -0.04 [-0.08 to 0.00]). Similarly, IV(+) did not significantly affect the difference in HR (-0.23 vs. 1.16; difference [95% CI]: -1.40 [-3.59 to 0.80]) or RR (-1.95 vs. -1.08; difference [95% CI]: -0.87 [-1.83 to 0.09]), both demonstrating small effect sizes. However, IV(+) was associated with a significant increase in the difference in sBP, although the effect size remained small (13.22 vs. 8.73; difference [95% CI]: 4.49 [0.35 to 8.62]).

**Table 2:** Results of significance tests with and without IV and results of supplemental analysis by IPW ^a^ n = 19,793; IV(+): n = 390, IV(-): n = 19,403 ^b ^n = 778; IV(+): n = 389, IV(-): n = 389 ^c^ IV(+): n = 19,733.3, IV(-): n = 19,803 ATE, average treatment effect; DSI, delta shock index; HR, heart rate; IPW, inverse probability weighting; IV, intravenous; RR, respiratory rate; sBP, systolic blood pressure

Endpoint	IV(+)			IV(-)			Difference (95%CI)	p-value	Cohen’s d
	Difference mean (SD)	Pre-hospital value mean	Arrival hospital value mean	Difference mean (SD)	Pre-hospital value mean	Arrival hospital value mean			
DSI									
Unadjusted^a^	-0.09 (0.29)	0.89	0.80	-0.02 (0.16)	0.64	0.62	-0.07 (-0.11, -0.05)	<0.0001	0.43
Adjusted^b^	-0.09 (0.29)	0.90	0.80	-0.06 (0.27)	0.87	0.81	-0.04 (-0.08, 0.00)	0.05	0.14
sBP difference (mmHg)									
Unadjusted^a^	13.29 (30.60)	107.16	120.45	1.01 (25.41)	141.21	142.23	12.27 (9.21, 15.35)	<0.0001	0.48
Adjusted^b^	13.22 (30.60)	107.22	120.43	8.73 (28.14)	112.90	121.63	4.49 (0.35, 8.62)	0.03	0.15
HR difference (beats/min)									
Unadjusted^a^	-0.18 (16.30)	89.19	89.00	-1.43 (12.11)	85.85	84.42	1.25 (-0.38, 2.88)	0.13	0.10
Adjusted^b^	-0.23 (16.29)	89.20	88.97	1.16 (14.90)	89.80	90.97	-1.40 (-3.59, 0.80)	0.21	0.09
RR difference (counts/min)									
Unadjusted^a^	-1.96 (6.72)	24.48	22.53	-0.90 (5.82)	21.40	20.49	-1.05 (-1.72, -0.38)	0.002	0.13
Adjusted^b^	-1.95 (6.73)	24.47	22.52	-1.08 (6.93)	23.35	22.28	-0.87 (-1.83, 0.09)	0.07	0.13
DSI (supplemental analysis)									
IPW (ATE)^c^	-0.03 (1.27)	-	-	-0.02 (0.17)	-	-	-0.02 (-0.04, 0.00)	0.07	0.02

**Figure 3:**
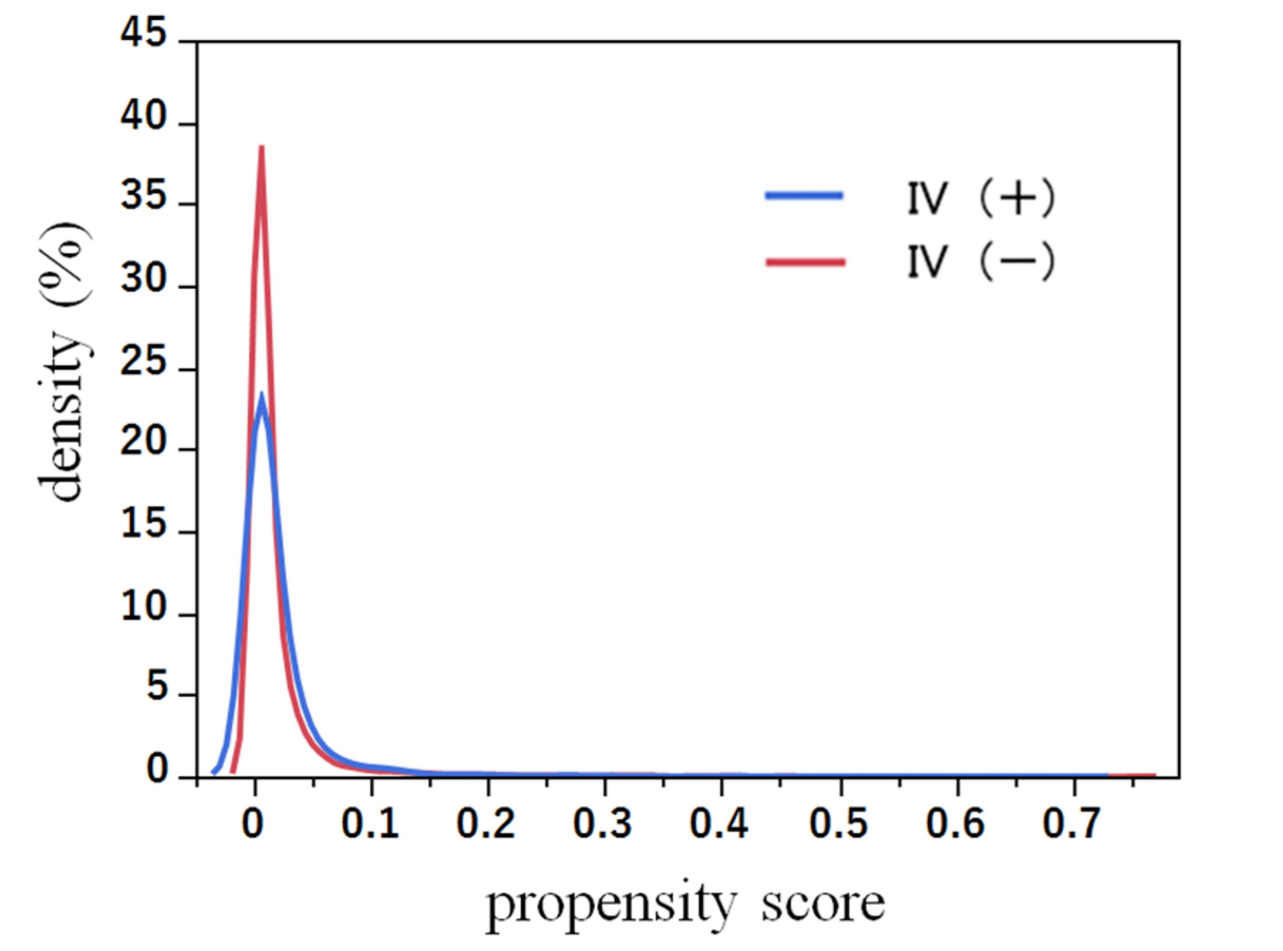
Supplemental analysis: comparison of PS densities for pre-hospital IV after IPW analysis ATE, average treatment effect; IPW, inverse probability weighting; IV, intravenous; PS, propensity score ^a^ IV(+) n = 18,733.3, IV(-) n = 19,803.6

After applying IPW adjustment, there were 18,733.3 patients in the IV(+) group and 19,803.6 patients in the IV(-) group eligible for analysis. The PS density and SMDs were confirmed, indicating that a balance between the two groups was achieved (Table 3, Figure 3). Table 2 presents the IPW-adjusted results, showing that IV(+) was not significantly associated with changes in the DSI, with a small effect size observed (-0.03 vs. -0.02; difference [95% CI]: -0.02 [-0.04 to 0.00]).

**Table 3:** Supplemental analysis: patient characteristics with and without pre-hospital IV after IPW ATE, average treatment effect; ELST, emergency life-saving technician; HR, heart rate; IPW, inverse probability weighting; ISS, injury severity score; IV, intravenous; JCS, Japan Coma Scale; RR, respiratory rate; sBP, systolic blood pressure; SMD, standardized mean difference

Variables	After IPW (ATE)				
	IV(+)		IV(-)		SMD
	n = 18,733.3 (%)		n = 19,803.6 (%)		
Age					0.043
15-64	9,385.40	(50.10)	9,070.00	(45.80)	
≥65	9,347.90	(49.90)	10,733.60	(54.20)	
Sex: male	11,745.80	(62.70)	11,921.80	(60.20)	0.025
Trauma classification					0.005
Blunt injury	18,152.60	(96.90)	19,090.70	(96.40)	
Penetrating injury	580.70	(3.10)	712.90	(3.60)	
Mechanism of injury					0.011
Traffic accident	6,706.50	(35.80)	7,426.40	(37.50)	
Fall	10,190.90	(54.40)	10,674.10	(53.90)	
Other	1,835.90	(9.80)	1,703.10	(8.60)	
Transport time (min)					0.035
1-9	7,474.60	(39.90)	7,446.20	(37.60)	
10-15	6,200.70	(33.10)	5,960.90	(30.10)	
16-61	5,058.00	(27.00)	6,396.60	(32.30)	
Oxygen inhalation by ELST	8,504.90	(45.40)	8,495.70	(42.90)	0.024
Spinal motion restriction by ELST	9,984.80	(53.30)	9,802.80	(49.50)	0.038
Hemostasis and fixed by ELST	2,510.30	(13.40)	2,119.00	(10.70)	0.027
Airway management by ELST	187.30	(1.00)	217.80	(1.10)	<0.001
Pre-hospital sBP (mmHg)					0.009
60-89	1,086.50	(5.80)	911.00	(4.60)	
90-199	17,178.40	(91.70)	18,120.30	(91.50)	
200-240	468.30	(2.50)	772.30	(3.90)	
Pre-hospital HR (beats/min)					0.003
40-49	37.50	(0.20)	99.00	(0.50)	
50-119	17,684.20	(94.40)	18,615.40	(94.00)	
120-150	1,011.60	(5.40)	1,089.20	(5.50)	
Pre-hospital RR (counts/min)					0.025
10-29	16,504.00	(88.10)	17,942.10	(90.60)	
30-50	2,229.30	(11.90)	1,861.50	(9.40)	
Pre-hospital JCS					0.014
0	9,741.30	(52.00)	10,040.40	(50.70)	
1-3	6,706.50	(35.80)	7,366.90	(37.20)	
10-30	1,161.50	(6.20)	1,247.60	(6.30)	
100-300	1,124.00	(6.00)	1,128.80	(5.70)	
Pre-hospital oxygen saturation (%)					0.008
60-89	1,067.80	(5.70)	970.40	(4.90)	
90-100	17,665.50	(94.30)	18,833.20	(95.10)	
Pre-hospital shock index					0.03
<1.0	16,972.40	(90.60)	18,516.40	(93.50)	
≥1.0	1,760.90	(9.40)	1,287.20	(6.50)	
ISS: ≥15	5,095.50	(27.20)	5,703.40	(28.80)	0.016
Head injury	7,062.50	(37.70)	6,099.50	(30.80)	0.069
Face injury	3,053.50	(16.30)	3,386.40	(17.10)	0.008
Neck injury	131.10	(0.70)	297.10	(1.50)	0.007
Thorax injury	5,844.80	(31.20)	5,842.10	(29.50)	0.017
Abdomen and pelvis injury	1,498.70	(8.00)	1,821.90	(9.20)	0.012
Spine injury	4,514.70	(24.10)	5,307.40	(26.80)	0.028
Upper extremity injury	6,125.80	(32.70)	5,208.30	(26.30)	0.064
Lower extremity injury	8,392.50	(44.80)	9,723.60	(49.10)	0.043
Body surface injury	2,004.50	(10.70)	891.20	(4.50)	0.061

## Discussion

This study demonstrated that IV administration did not significantly impact the trauma patients’ condition and physiological parameters in the pre-hospital setting, as all endpoint effect sizes were small and exhibited minimal variation. Specifically, IV administration resulted in a slight improvement in the shock index of -0.09 by the time of hospital arrival, a trend consistent with the findings from the IPW analysis. Furthermore, sBP increased by an average of 13.22 mmHg, with a significant difference of 4.49 mmHg compared to cases without IV. However, the effect size was small and did not lead to extreme fluctuations in the DSI, sBP, HR, or RR.

The observed increase in sBP, even in the absence of IV, suggests that the body’s compensatory mechanisms may have been activated due to biological homeostasis. Factors such as catecholamines, antidiuretic hormone, and atrial natriuretic peptides may have facilitated vasoconstriction and increased cardiac output, leading to elevated sBP without IV administration [25,26].

Shock, particularly due to traumatic injury, typically arises from a reduction in blood volume to tissues and organs, compromising normal cellular function. It becomes life-threatening when average blood pressure drops below 60 mmHg, impeding adequate blood flow to vital organs [3,20]. Therefore, improving circulatory dynamics by replenishing deficient extracellular fluid to maintain organ perfusion is crucial. However, pre-hospital IV therapy aimed at replenishing extracellular fluid poses challenges, as excessive infusion may exacerbate dilutional coagulopathy. The global trend is moving toward restricted use of crystalloids for traumatic hemorrhagic shock [27].

Japanese ELSTs, operating in the pre-hospital setting, have a limited range of interventions available for shock, including IV administration, oxygen inhalation for cellular hypoxemia, and positioning patients with elevated lower extremities to enhance tissue perfusion to vital organs. In this context, pre-hospital IV may serve as a valuable treatment to stabilize patients during transport to the hospital without exacerbating their condition, aligning with the mission of Japanese ELSTs to prevent deterioration and ensure safe transport.

Pre-hospital IV administration, guided remotely online, varies in timing, volume, and rate according to specific pathologies, such as severe trauma. Compared to cases without IV, the administration by ELSTs, under medical supervision, may be clinically beneficial from a telemedicine perspective. Although this study does not specifically address the infusion volume and analyzed a limited number of eligible patients, it contributes to understanding the extent and impact of IV on physiological parameters in the pre-hospital setting.

The novelty and strength of this study lie in its focus on analyzing the effect of IV on fluctuations in physiological parameters, particularly in the pre-hospital context. Since IV therapy is a symptomatic treatment, examining the fluctuations of these parameters up to hospital arrival, rather than solely focusing on outcomes, may provide valuable insights for ELSTs and paramedics operating in the pre-hospital environment.

Limitations

This study encountered several limitations. First, as it utilized secondary data from the JTDB, critical variables such as the volume, rate of infusion, and timing of IV implementation were not recorded [5]. Second, employing listwise deletion to handle missing data (23,659 patients, representing 26.6% of the dataset) and excluding cases that did not meet eligibility criteria (45,365 patients, representing 51.1% of the dataset) may have introduced bias into the estimates [28]. Third, the specific fire department that performed the procedures was not identified, and IV protocols varied across different regional MC jurisdictions. Fourth, the JTDB might have included patients with cardiogenic shock - typically not indicated for IV therapy - due to their trauma-related injuries. Fifth, the entire sample could not be analyzed since the analysis was limited to the PSM sample, which excluded the off-support population, thereby reducing the generalizability of the findings. In a supplementary analysis, however, the IPW-adjusted analysis did include the off-support population. Finally, extreme vital sign values were removed to eliminate large fluctuations (totaling 778 cases, or 0.9%); nonetheless, this did not introduce significant selection bias.

## Conclusions

We utilized the nationwide JTDB to examine the impact of IV therapy administered by ELSTs on trauma patients in the pre-hospital setting. Our findings indicated that IV therapy was not directly associated with variations in the shock index; however, it significantly increased sBP. Future studies should focus on incorporating the volume of IV infusion to provide a more comprehensive understanding of its effects.
